# Preparation and Characterization of Hyaluronic Acid-Polycaprolactone Copolymer Micelles for the Drug Delivery of Radioactive Iodine-131 Labeled Lipiodol

**DOI:** 10.1155/2017/4051763

**Published:** 2017-01-03

**Authors:** Shih-Cheng Chen, Ming-Hui Yang, Tze-Wen Chung, Ting-Syuan Jhuang, Jean-Dean Yang, Ko-Chin Chen, Wan-Jou Chen, Ying-Fong Huang, Shiang-Bin Jong, Wan-Chi Tsai, Po-Chiao Lin, Yu-Chang Tyan

**Affiliations:** ^1^Office of Research and Development, Kaohsiung Medical University, Kaohsiung 807, Taiwan; ^2^Center for Infectious Disease and Cancer Research, Kaohsiung Medical University, Kaohsiung 807, Taiwan; ^3^Department of Biomedical Engineering, National Yang-Ming University, Taipei 112, Taiwan; ^4^Invasive Cardiac Laboratory, Tan Tock Seng Hospital, Singapore 308433; ^5^Department of Chemistry, Chung Yuan Christian University, Taoyuan 320, Taiwan; ^6^Department of Pathology, Changhua Christian Hospital, Changhua 500, Taiwan; ^7^Department of Medical Imaging and Radiological Sciences, Kaohsiung Medical University, Kaohsiung 807, Taiwan; ^8^Department of Nuclear Medicine, Kaohsiung Medical University Hospital, Kaohsiung 807, Taiwan; ^9^Department of Medical Laboratory Science and Biotechnology, Kaohsiung Medical University, Kaohsiung 807, Taiwan; ^10^Department of Laboratory Medicine, Kaohsiung Medical University Hospital, Kaohsiung 807, Taiwan; ^11^Department of Chemistry, National Sun Yat-sen University, Kaohsiung 804, Taiwan; ^12^Graduate Institute of Medicine, College of Medicine, Kaohsiung Medical University, Kaohsiung 807, Taiwan; ^13^Institute of Medical Science and Technology, National Sun Yat-sen University, Kaohsiung 804, Taiwan; ^14^Department of Medical Research, Kaohsiung Medical University Hospital, Kaohsiung 807, Taiwan

## Abstract

Micelles, with the structure of amphiphilic molecules including a hydrophilic head and a hydrophobic tail, are recently developed as nanocarriers for the delivery of drugs with poor solubility. In addition, micelles have shown many advantages, such as enhanced permeation and retention (EPR) effects, prolonged circulation times, and increased endocytosis through surface modification. In this study, we measured the critical micelle concentrations, diameters, stability, and cytotoxicity and the cell uptake of micelles against hepatic cells with two kinds of hydrophilic materials: PEG-PCL and HA-g-PCL. We used ^131^I as a radioactive tracer to evaluate the stability, drug delivery, and cell uptake activity of the micelles. The results showed that HA-g-PCL micelles exhibited higher drug encapsulation efficiency and stability in aqueous solutions. In addition, the ^131^I-lipiodol loaded HA-g-PCL micelles had better affinity and higher cytotoxicity compared to HepG2 cells.

## 1. Introduction

The iodized ester of poppy-seed oil, lipiodol, has been widely used as an iodine-based contrast agent for better radiological imaging, which enhances radiopaque contrast of the examined organs and/or tissues [1]. Lipiodol was further found to be mainly retained in the liver after arterial injection and selectively localized in hepatocellular carcinoma tissues [2–4]. It was generally applied as an embolization agent to treat hepatic carcinoma and as a carrier to selectively deliver conjunctional antitumor molecules [5–11]. Recently, lipiodol labeled with radioactive ^131^iodine (^131^I) has been further applied as a radioactive agent to treat hepatocellular carcinoma [12–14].

Although it has been shown that more than 75% ^131^I-lipiodol was retained in liver following arterial administration [2], the remains of injected ^131^I-lipiodol circulating elsewhere in the body potentially cause severe off-target complication, for example, pneumopathies and hypothyroidism. Thus, a novel delivery system that targets ^131^I-lipiodol specifically to tumor cells is highly anticipated. By targeting ^131^I-lipiodol specifically to tumorous cells not only can the potential off-target complication be reduced in nonliver tissues, but also the efficacy of destroying solid liver tumors can be improved with the highly localized antitumor and/or radioactivity.

Hyaluronic acid (HA), the polydisaccharides composed of D-glucuronic acid and D-N-acetylglucosamine, is one of the major components of extracellular matrix in connective tissues. The HA polymers in vivo can range from 5,000 to 20,000,000 Daltons with 2,000 to 25,000 disaccharide repeats in length [15, 16]. Recently, HA has been shown to be one of the key modulators in various human tumors, in terms of affecting cell proliferation rate, changing cellular motility, controlling the malignity, and mediating angiogenesis [17, 18]. In many types of tumors, the HA receptors, for example, CD44 and receptor for HA-mediated motility (RHAMM), are highly expressed and activated, and hence the cell infiltration and tumor malignity are promoted [19, 20]. Recently, it has been reported that HA conjugation promotes the uptake of conjugated particles with the carried agents in HepG2 cells [21–23].

In this report, we demonstrated the preparation of ^131^I-lipiodol loaded micelles made of polyethylene glycol-polycaprolactone (PEG-PCL) copolymer and HA-g-PCL amphiphilic copolymers. And the critical micelle concentration, encapsulation efficiency, particle size, and micelle stability of the two amphiphilic copolymers were characterized. Furthermore, the cytotoxic and cell uptake of ^131^I-lipiodol loaded micelles in hepatocellular carcinoma cells was confirmed and the potential of ^131^iodine-labeled lipiodol loaded HA-g-PCL micelles for radio/embolic therapy was discussed.

## 2. Materials and Methods

### 2.1. Isotopic Exchange Labeling of Lipiodol with ^131^Iodine

The radioactive ^131^I was used to label lipiodol with the procedure adapted from the method described by Lo et al., 1992 [24]. In brief, 0.5 mCi radioactive NaI (Global Medical Solutions, Taiwan) in 1 mL ethanol was mixed and incubated at 80°C for 30 min with 1 mL lipiodol (Guerbet, USA), followed by heating up to 100°C for 30 min to remove residual ethanol and water. The radioactivity of ^131^I-lipoidol was subjected to thin-layer chromatography (TLC) to understand the labeling efficiency.

### 2.2. Measurement of Critical Micelle Concentration

**Figure 1:**
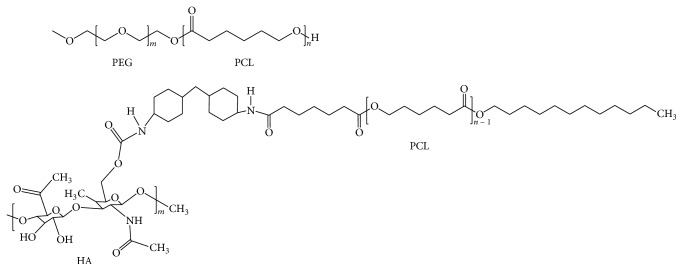
The chemical structures of PEG-PCL and HA-g-PCL monomers.

Two different amphiphilic copolymers, PEG-PCL and HA-g-PCL (Figure 1), were precious gifts provided by Dr. Yang from Industrial Technology Research Institute, Taiwan. The critical micelle concentrations of the two copolymers were measured by pyrene-based fluorescent probe method reported by Kalyanasundaram and Thomas, 1977, and Aguiar et al., 2003 [25, 26]. The PEG-PCL and HA-g-PCL amphiphilic copolymers were serially diluted with ultrapure water in 16 test tubes to the final concentration ranging from 1.0 mg/mL to 6.0 × 10^−5^ mg/mL, respectively. Subsequently, pyrene was added to each vial with gentle agitation to the final concentration of 0.6 *μ*M, and all the test tubes were incubated overnight (16 hours) without exposure to light. Applying excitation wavelength of 270–360 nm, the ratio between the emission intensity at 337 nm and 334 nm was plotted against the base 10 logarithm of the concentration of each tested sample to determine the CMC of each copolymer.

### 2.3. Preparation of ^131^I-Lipiodol Containing Micelles

Approximately 2.4 mg of PEG-PCL or HA-g-PCL copolymers was dissolved in 2 mL DMSO by sonication for 20 min, followed by adding 10 *μ*L ^131^I-lipiodol with sonication at room temperature (20°C) for 5 minutes, respectively. Then the two homogenized ^131^I-lipiodol copolymer DMSO solutions were sonicated for 20 min, stirred for 30 min, and dialyzed with 5-liter ultrapure water at room temperature for 12 hours, respectively, of which the dialyzing ultrapure water was refreshed after 1 and 4 hours. After the 12-hour dialysis, ^131^I-lipiodol loaded micelles were formed and suspended in ultrapure water. The protocol for lipophilic molecules was conducted according to standard procedures [27].

### 2.4. Cell Culture

Liver cells (CCL-13) and liver tumor cells (HepG2) were cultured and maintained as previously reported [28]. In general, cells were maintained in Dulbecco's Modified Eagle's Medium (DMEM) with 10% Fetal Bovine Serum (FBS) at 37°C, 95% related humidity, and 5% CO_2_.

### 2.5. Measurement of ^131^I-Lipiodol Uptakes in Cells

Seven hundred thousand CCL-13 and HepG2 cells were seeded in 10 cm dishes and cultured for 48 hours, respectively, prior to the treatment of ^131^I-lipiodol loaded micelles (0.5 mCi/day). Eight hundred microliters of freshly made micelles was added to the cell containing culture dishes and incubated at 37°C, 95% RH, and 5% CO_2_. After 6, 24, and 48 hours, the cells were collected and washed with PBS and subjected to the measurement of cell contained radioactivity with a well-type scintillator, respectively, which represented the cell uptake of ^131^I-lipiodol delivered by PEG-PCL and HA-g-PCL micelles. The cell cytotoxicity study was determined with the lactate dehydrogenase leakage (LDH) assay leakage into the culture medium. The LDH assay is based on the conversion of lactate into pyruvate in the presence of LDH with a parallel reduction of NAD. The formation of NADH from the above reaction results in a change in absorbance at 340 nm.

## 3. Result and Discussion

### 3.1. The Critical Micelle Concentrations of HA-g-PCL and PEG-PCL Copolymers

**Figure 2:**
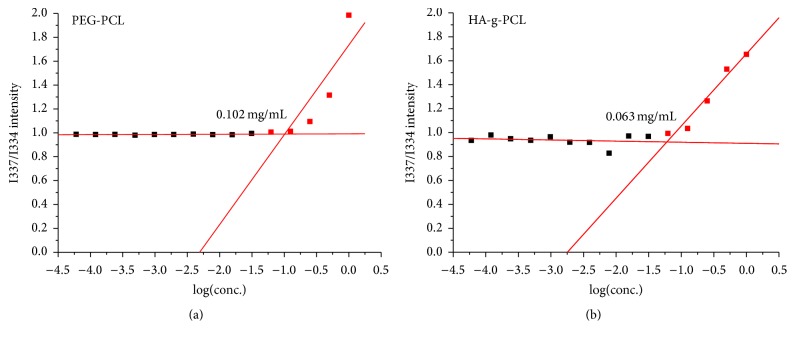
The critical micelle concentrations of PEG-PCL (a) and HA-g-PCL (b) copolymers. It has been suggested that the critical micelle concentration is negatively correlated to the stability of forming micelles in general.

Using pyrene-based fluorescent probe method, the critical micelle concentrations of PEG-PCL and HA-g-PCL amphiphilic copolymers were calculated 0.102 mg/mL and 0.063 mg/mL, respectively, according to the intensity ratio of 337/334 nm plotted against the base 10 logarithm of the tested concentration series (Figure 2). It has been suggested that the critical micelle concentration is negatively correlated to the stability of forming micelles in general [29, 30], that is, the lower the critical micelle concentration that the copolymer has, the more the micelles form in a stable way. In a sense, low critical micelle concentration implies the greater resistance to disruption caused by the rapid dilution effect after intravenous or arterial injection. Therefore, HA-g-PCL copolymer can be a suitable copolymer for drug-load delivering for its low critical micelle concentration.

### 3.2. The Encapsulation Efficiency of HA-g-PCL and PEG-PCL Copolymers

The lipiodol was labeled using isotopic exchanging method [24] in this study, of which more than 95% iodine of the lipiodol has been exchanged to the radioactive ^131^I according to TLC analysis and the measurement of relative radioactivity. Taking the advantage of the autoradioactivity of ^131^I-lipiodol, we were able to calculate the encapsulation efficiency and the yield of loaded micelles by tracking the radioactivity without disrupting the micelles. The encapsulation efficiency was determined by comparing the radioactivity of ^131^I-lipiodol-copolymer mix that remained after dialysis with the total input prior to dialysis. The encapsulation efficiencies of PEG-PCL and HA-g-PCL copolymers were 69.16 ± 1.84% and 71.00 ± 1.76%, respectively, showing that both copolymers encapsulate ^131^I-lipiodol well (Table 1). These encapsulation efficiencies are better than some of the recently reported PCL-based copolymers for drug-load which ranged from approximately 15% to 60% [31–33]. The high encapsulation efficiency suggested that HA-g-PCL is a promising copolymer for ^131^I-lipiodol.

### 3.3. The Yield of ^131^I-Lipiodol Loaded HA-g-PCL and PEG-PCL Micelles

The yield rates of micelles made of HA-g-PCL and PEG-PCL copolymers are significantly different, though the encapsulation efficiencies of the two copolymers are more or less the same. By tracking the radioactivity, only 17.93 ± 0.70% of ^131^I-lipiodol loaded PEG-PCL micelles could pass through the filtration with 450 nm cutoff, suggesting that the original particle sizes of the ^131^I-lipiodol loaded PEG-PCL micelles were larger than 450 nm. Thus, the encapsulation efficiencies of both kinds of micelles were similar; however, the filtration rate of ^131^I-lipiodol loaded HA-g-PCL micelles was much better than that of the ^131^I-lipiodol loaded PEG-PCL micelles. Thus, 63.03 ± 1.43% of ^131^I-lipiodol loaded HA-g-PCL micelles passed through the filter. As a result, the yield rate of HA-g-PCL micelles is much higher than that of PEG-PCL, which was around 44.76 ± 1.50% comparing with 12.40 ± 0.59%, respectively (Table 1). Thus, HA-g-PCL copolymer is suitable to generate ^131^I-lipiodol loaded micelles with good yield.

### 3.4. The Particle Size of ^131^I-Lipiodol Loaded Micelles

**Figure 3:**
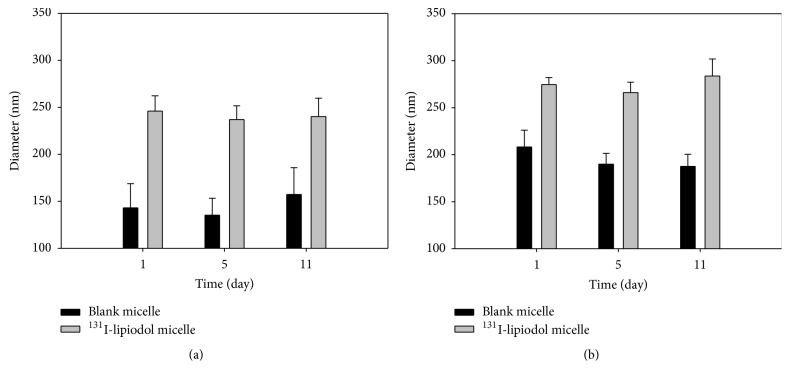
The particle size of ^131^I-lipiodol loaded micelles. As determined by particle size analyzer, the average size of the ^131^I-lipiodol loaded micelles in day 1 were 246 ± 16 nm ((a) PEG-PCL) and  274 ± 4 nm ((b) HA-g-PCL) in the phosphate-buffered saline, respectively.

After passing through the 0.45 *μ*m filter, the particle sizes of micelles made of HA-g-PCL and PEG-PCL amphiphilic copolymers were measured with particle analyzer, Zetasizer 3000 HSA, (Malvern Instruments, UK).  Figure 3 showed the particle sizes of ^131^I-lipiodol loaded and nonloaded micelles. Due to the fact that HA-g-PCL monomer molecular size and weight were higher than those of the PEG-PCL monomer, the particle sizes of nonloaded HA-g-PCL micelles were around 210 nm in diameter, which were larger compared to the PEG-PCL micelles (150 nm). The fresh ^131^I-lipiodol loaded HA-g-PCL micelles were on average 274 ± 4 nm in diameter (Figure 3(b)). The particle size of HA-g-PCL micelles did not significantly change over time, showing similar particle sizes after 4 and 10 days. Nevertheless, there was a trend observed that the particle size of nonloaded micelles seemed to be reduced slightly after 4 days; yet it kept constant till the 10th day. Thus, the loading of ^131^I-lipiodol not only resulted in a larger particle size to be formed with HA-g-PCL copolymer but also resulted in a better constancy of the particles size over time. Similar trend accounts for the particles made of PEG-PCL copolymers that the ^131^I-lipiodol loaded micelles were 246 ± 16 nm in diameter while the nonloaded particles were 130 to 160 nm in diameter with high variation over time.

### 3.5. The Stability of ^131^I-Lipiodol Loaded Micelles

**Figure 4:**
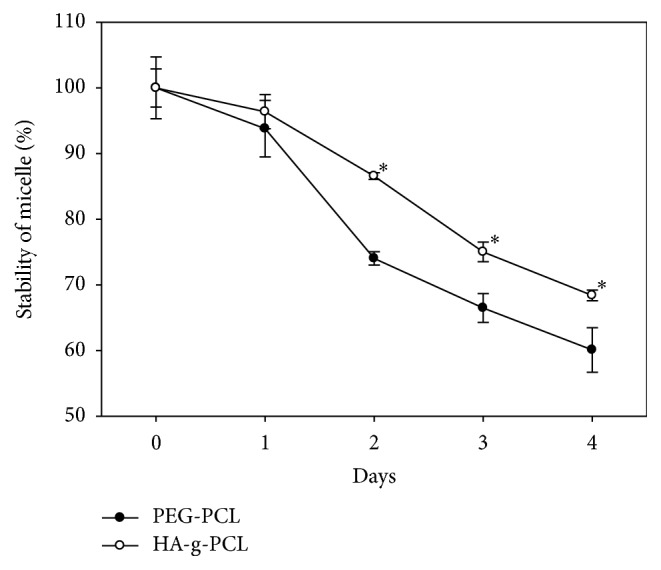
The stability of ^131^I-lipiodol loaded micelles. In this study, it was shown that both ^131^I-lipiodol loaded HA-g-PCL and PEG-PCL micelles kept their integrities well, holding as high as 60%  ^131^I-lipiodol in the micelles after 4 days (*n* = 10, ^*∗*^*p* < 0.05).

By monitoring the radioactivity kept encapsulating in the micelles under continuous dialysis, the stability of micelles can be evaluated over time. The stability, in terms of keeping the integrity of micelles without releasing the encapsulated ^131^I-lipiodol to the dialyzing ultrapure water, of ^131^I-lipiodol loaded HA-g-PCL and PEG-PCL copolymers over time was shown in Figure 4. It was shown that both ^131^I-lipiodol loaded HA-g-PCL and PEG-PCL micelles kept their integrities well, holding as high as 60%  ^131^I-lipiodol in the micelles after 4 days. Comparing with other recently reported PCL-base micelles [23, 34–36], we found that HA-g-PCL copolymer encapsulated ^131^I-lipiodol well and showed relatively low leakage of the micelles loaded with ^131^I-lipiodol over time in vitro. On the other hand, HA-g-PCL copolymers also showed low leaking encapsulation of ^131^I-lipiodol in our study, which was superior to other recently reported HA-based micelles/nanoparticles for differential drug deliveries [37–40]. Thus, HA-g-PCL copolymers and ^131^I-lipiodol may be a particular suitable carrier-drug pair; for example, the HA-g-PCL copolymers are by nature a good micellar carrier for the ^131^I-lipiodol while the loading of ^131^I-lipiodol potentially upholds the stability of the formed micelles. These characteristics make HA-g-PCL micelles an ideal delivery system for ^131^I-lipiodol.

### 3.6. Cell Uptake and Cytotoxicity of Micelles Encapsulated with ^131^I-Lipiodol

**Figure 5:**
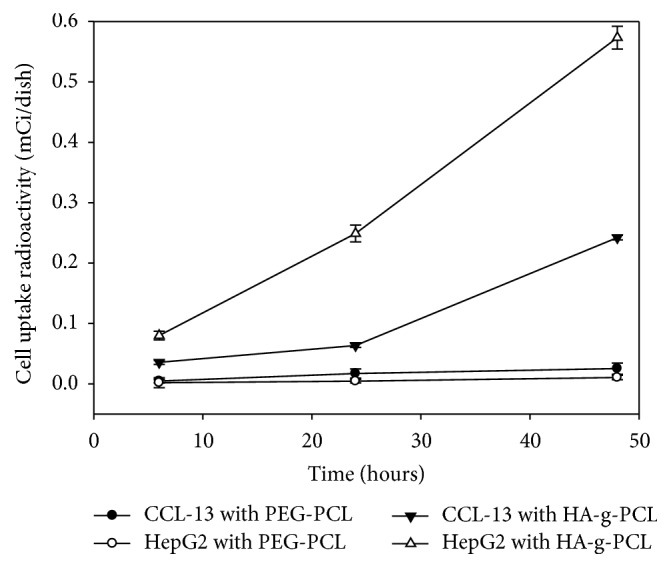
The cell uptake of the micelles encapsulated with ^131^I-lipiodol. The HepG2 showed better uptake of ^131^iodine-labeled lipiodol delivered by HA-g-PCL micelles than did CCL-13 cells.

When cells were treated with ^131^I-lipiodol micelles, a direct way to know the cell uptake level of ^131^I-lipiodol is to monitor the intracellular radioactivity after the treatment.  Figure 5 showed the proportion of intracellular ^131^I-lipiodol detected over time in CCL-13 and HepG2 cells. The ^131^I-lipiodol loaded HA-g-PCL micelles delivered 0.58 mCi loaded ^131^I-lipiodol to HepG2 cells 48 h after the treatment but delivered only 0.24 mCi to CCL-13 cells under the same condition (Figure 5). One of the reasons responsible for the differential ^131^I-lipiodol uptake between HepG2 and CCL-13 cells may be that the superficial HA conjugation of the HA-g-PCL micelles promotes a selective uptake mediated by HA receptors in HepG2 cells. Previously we have reported that HepG2 cells express high levels of CD44 particularly after treatment of chitosan nanoparticles, while such differential expression of CD44 was not found in the CCL-13 cell [28]. Since CD44 is one of the major HA receptors, it is likely that the CD44 abundant HepG2 cells allowed the superficial conjugated HA on HA-g-PCL micelles to selectively interact with their HA receptors located on the cell surface. As a result, the HepG2 showed better uptake of ^131^iodine-labeled lipiodol delivered by HA-g-PCL micelles than did CCL-13 cells.

In the experiment of delivering ^131^I-lipiodol with PEG-PCL copolymers, we found that the cell uptakes in both CCL-13 and HepG2 cells were much lower compared to that delivered with HA-g-PCL copolymers (Figure 5). Clearly, PEG-PCL had lower delivery efficiency of ^131^I-lipiodol than did HA-g-PCL, probably due to the differential properties between PEG- and HA-conjugated micelles, for example, the particle size and charges,. Since there was no superficial HA conjugation on ^131^I-lipiodol loaded PEG-PCL micelles, the PEG-PCL copolymers did not show HA-mediated selectivity. Notably, more ^131^I-lipiodol was found to accumulate in CCL-13 cells compared to Hep2G cells when delivered with PEG-PCL copolymers. We have found that approximately 2-fold ^131^I-lipiodol was accumulated in CCL-13 compared to HepG2 cells after 48 hours of treatment. It is difficult to identify a sole reason for such differential uptake, but some possibilities may be speculated. There may be HA-independent mechanism of micelle uptake and/or cytotoxicity that promotes the ^131^I-lipiodol accumulation and/or survival of CCL-13 cells over Hep2G cells, and this preference becomes more significant when a highly efficient carrier was introduced. However, because the dosages of cell uptake were very low, there is no significant difference between CCL-13 and Hep2G cells.

**Figure 6:**
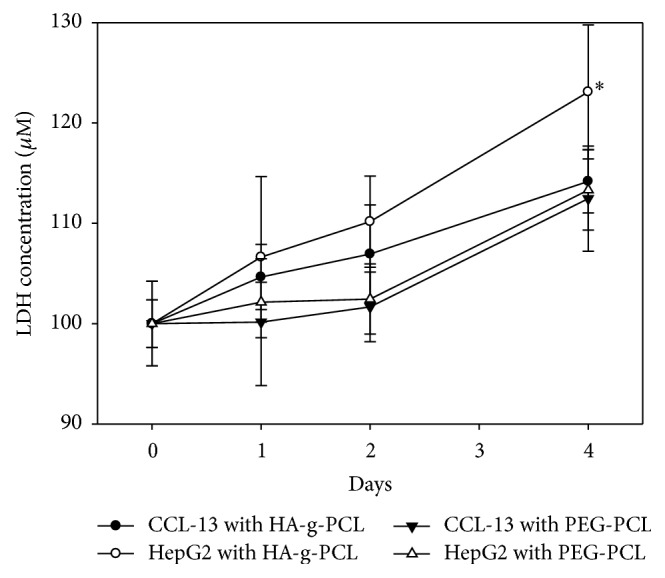
The cytotoxicity of the micelles encapsulated with ^131^I-lipiodol. After 4 days of incubation, the LDH concentrations of HepG2 cells were increased significantly. It may be due to the increased cell uptake activity and higher stability of ^131^I-lipiodol loaded HA-g-PCL micelles which contributed to having better ^131^I-lipiodol control release rates (*n* = 10, ^*∗*^*p* < 0.05).

In this study, the concentrations of HA-g-PCL micelles in HepG2 were higher than those of PEG-PCL micelles in HepG2 cells. Additionally, the uptake of HA-g-PCL micelles in HepG2 cells was better than that in CCL-13 cells. HepG2 cells, expressing CD44 and RHAMM abundantly and having HA receptors, showed a higher uptake rate compared to ^131^I-lipiodol loaded HA-g-PCL micelles. Thus, it showed the potential of applying HA-g-PCL micelles for targeted delivery of radioactive ^131^I-lipiodol, which may lead to a breakthrough of radiochemotherapy. The cytotoxicity study demonstrated most ^131^I-lipiodol loaded micelles were low cytotoxic or noncytotoxic. However, after 4 days of incubation with ^131^I-lipiodol loaded HA-g-PCL micelles, the LDH concentrations of HepG2 cells were increased significantly (Figure 6). It may be due to the increased cell uptake activity and higher stability of ^131^I-lipiodol loaded HA-g-PCL micelles which contributed to having better ^131^I-lipiodol control release rates. In addition, the HepG2 cells absorbed higher radiation doses and may cause internal cell damage. The HepG2 cells were hepatic tumor cells, and, based on our experimental results, ^131^I-lipiodol loaded HA-g-PCL micelles may show the avenue for possible target radiotherapy. The well-studied PCL-based material is particularly suitable for the development of drug delivery and for that its biosafety was proven. Targeted delivery of radioactive agents is a challenging field which is yet of urgent importance for clinical application. Indeed, the studies of drug absorption, distribution, metabolism, and excretion are important and the biochemical and physiologic effects of drugs should be illustrated.

## 4. Conclusion

The amphiphilic HA-g-PCL copolymer is a potent carrier for lipiodol. It can form lipiodol loaded micelles, which are 270 to 280 nm in diameter. By labeling lipiodol with ^131^I, the loaded lipiodol can be tracked by the radioactivity. We have shown that the micelles were highly stable in vitro and selectively deliver more loads to HepG2 cell which is hepatocellular carcinoma cell line previously reported to express high levels of CD44. Thus the HA-g-PCL copolymer is an ideal carrier of lipiodol. On the other hand, not only did the radioactivity of ^131^I-lipiodol allow us to study the uptake of targeted cells in vitro but also it has potential to be applied as a targeted radiotherapy. It may be worth focusing on studying the efficacy and the selectivity of ^131^I-lipiodol loaded HA-g-PCL micelles in hepatocellular carcinoma animal models and ultimately bringing the treatment to clinical trials in the future.

## Figures and Tables

**Table 1:** The final yield of the ^131^I-lipiodol loaded micelles by tracking the radioactivity.

	PEG-PCL	HA-g-PCL
Encapsulate efficiency	69.16 ± 1.84	71.00 ± 1.76
Filtration rate	17.93 ± 0.70	63.03 ± 1.43
Final yield	12.40 ± 0.59	44.76 ± 1.50

The encapsulation efficiency is the percentage of radioactivity before and after dialysis; the filtration rate is the percentage of radioactivity before and after the usage of a 0.45 *μ*m filter.
